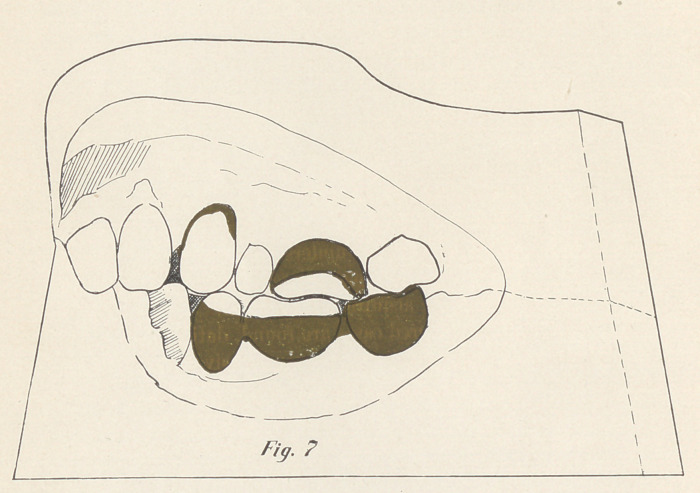# A Suspension Crown

**Published:** 1900-05

**Authors:** Horatio C. Meriam

**Affiliations:** Salem, Mass.


### International Dental Journal.

Vol. XXI. May, 1900. No. 5.

### Original Communications.[1]

### A SUSPENSION CROWN.[2]

BY HORATIO C. MERIAM, SALEM, MASS.

Besides the personal advantage gained by reporting cases like the one I bring before you to-night, there is also the satisfaction that we are making a record that may be of more value in the future than can be measured at the present. There can hardly be better evidence than the files of scientific journals, and by making our journal one of record we are recording now evidence that may in the future aid some dentist in his fight with the companies that will be formed on devices and operations. Some years since, Dr. Rollins, of Boston, in an article on porcelain inlays, gave a cut and a description of a bur made for him as ordered. He little thought, perhaps, in printing this in a medical journal, that some years after the record there made would be evidence sufficient to decide the right to make instruments. Had editors of that day kept in touch with correlated scientific journals, and made their journals journals of record for our advantage, they would have figured the new in-

[1] The editor and publishers are not responsible for the views of authors of papers published in this department, nor for any claim to novelty, or otherwise, that may be made by them. No papers will be received for this department that have appeared in any other journal published in the country.

[2] Read before The New York Institute of Stomatology, February 6, 1900.

vention and so saved instrument-makers from loss in entering upon vexatious law-suits, in ignorance that the claim had been invalidated by previous invention and record.

It is pleasant to think that a Harvard graduate has given us improvements free of patents, if another Harvard graduate has helped to form the Tooth-Crown Company.

A short time since I was called to treat the case shown (Fig. 1). A first bicuspid had split, and much absorption of the alveolar had followed, which shows perhaps more plainly in the palatal position of the cast. An extension crown was inserted,[1] but a new device was needed to guard against the working down of the first superior molar, and to aid the muscles of mastication to perform their function. I wished to make an appliance easily cleaned and one that could be made without cutting the molar or bicuspid. These teeth were banded and the band reinforced. Our president, Dr. Bogue, has kept the tipping of the molars that follows extraction and consequent loss of occlusion well before us. The tipping in this case was made use of, and part of the forward portion of the band covered to prevent its working down. In a subsequent case I made a strong nodule of gold here to occlude with the upper molar. The bicuspid had been previously cut away, but the mesial and distal points of the band were bent to partially cover. An impression was then taken with the bands in place. (Fig. 2.) A large countersunk molar was selected and ground to a taper. (Fig. 3.) A wide, tapering gold band was made for this tooth (Fig. 4); the band held upright on a lead anvil, the tooth was then placed in the band, protected by a piece of air-chamber tin, and driven to a fit. This can also be done by holding the tooth and band in a strip of the air-chamber tin folded. The whole rested on a block and the tooth struck into place. (Fig. 5.) (For making bands that are to be spread in this way, or that require fitting to teeth where there has been recession of the gum, a firm gold should be used, as a soft gold may not stand up firmly when the tooth is driven, nor hold an irregular line.) The tooth and band were then placed against the upper molar and the band waxed in position against the molar and bicuspid. (Fig. 6, *a* and *b*.)

The tooth was then removed and the band fastened in place on the cast by filling it with investing plaster, allowing this to

[1] See INTERNATIONAL DENTAL JOURNAL, March, 1899.

*Fig. 1*

*Fig. 3*

*Fig. 2*

*Fig. 5*

*Fig. 4*

*Fig. 6*

*Fig. 7*

run through and around its lower portions. (Fig. 6, *c.*) It was then lightly soldered to the other bands, removed from the cast, and the soldering completed, working on the underside. I have since removed from the cast after waxing, and invested the whole with the underside uppermost, showing only the wax, soldering but once.

The cap for holding the tooth is completed by soldering a small cap on its base. This little cap is easily made from a disk by striking with a round-headed punch into the lead anvil. The case was then ready for finishing and polishing.

The crown was set with oxyphosphate cement, and the bands filled with softened gutta-percha and forced into place. I have since varied the operation somewhat to suit different cases, but the essential principle is, I think, covered by what I have shown to-night. (Fig. 7.) You will see that all parts can be reached by a brush, and that it can be kept polished and clean by the patient.

Other specimens that I pass around may be of interest as showing the line of work, but the evening is too full to describe them.

## Figures and Tables

**Fig. 1 f1:**
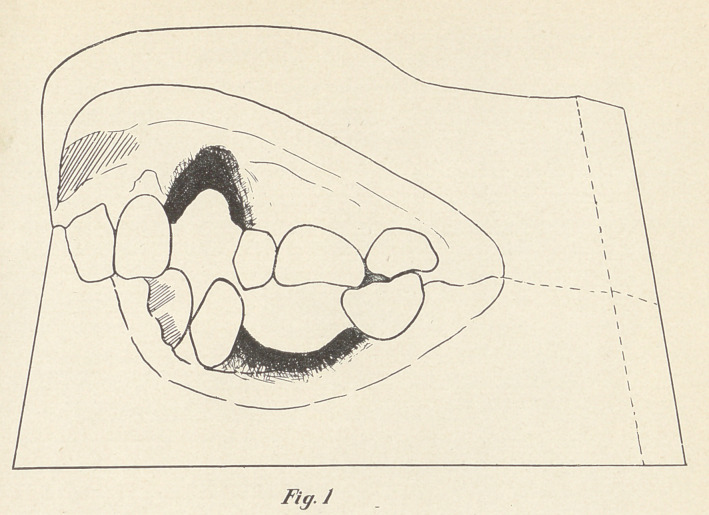


**Fig. 2 Fig. 3 Fig. 4 Fig. 5 f2:**
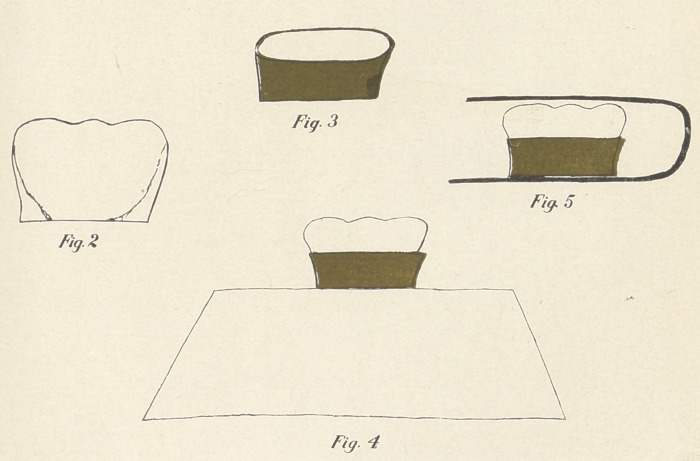


**Fig. 6 f3:**
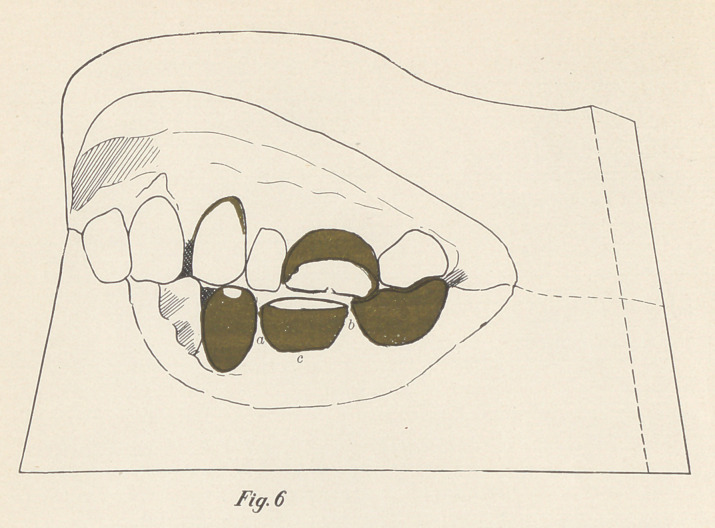


**Fig. 7 f4:**